# Vesicles From *Vibrio cholerae* Contain AT-Rich DNA and Shorter mRNAs That Do Not Correlate With Their Protein Products

**DOI:** 10.3389/fmicb.2019.02708

**Published:** 2019-11-22

**Authors:** Petter Langlete, Anders Kristian Krabberød, Hanne Cecilie Winther-Larsen

**Affiliations:** ^1^Section for Pharmacology and Pharmaceutical Biosciences, Department of Pharmacy, University of Oslo, Oslo, Norway; ^2^Centre for Integrative Microbial Evolution (CIME), Department of Biosciences, University of Oslo, Oslo, Norway; ^3^Section for Genetics and Evolutionary Biology (EVOGENE), Department of Biosciences, University of Oslo, Oslo, Norway

**Keywords:** *Vibrio cholerae*, extracellular vesicles, bacteriophages, RNA, DNA, extracellular proteomics

## Abstract

Extracellular vesicles secreted by Gram-negative bacteria have proven to be important in bacterial defense, communication and host–pathogen relationships. They resemble smaller versions of the bacterial mother cell, with similar contents of proteins, LPS, DNA, and RNA. Vesicles can elicit a protective immune response in a range of hosts, and as vaccine candidates, it is of interest to properly characterize their cargo. Genetic sequencing data is already available for vesicles from several bacterial strains, but it is not yet clear how the genetic makeup of vesicles differ from that of their parent cells, and which properties may characterize enriched genetic material. The present study provides evidence for DNA inside vesicles from *Vibrio cholerae* O395, and key characteristics of their genetic and proteomic content are compared to that of whole cells. DNA analysis reveals enrichment of fragments containing ToxR binding sites, as well as a positive correlation between AT-content and enrichment. Some mRNAs were highly enriched in the vesicle fraction, such as membrane protein genes *ompV, ompK*, and *ompU*, DNA-binding protein genes *hupA, hupB, ihfB, fis*, and *ssb*, and a negative correlation was found between mRNA enrichment and transcript length, suggesting mRNA inclusion in vesicles may be a size-dependent process. Certain non-coding and functional RNAs were found to be enriched, such as VrrA, GcvB, tmRNA, RNase P, CsrB2, and CsrB3. Mass spectrometry revealed enrichment of outer membrane proteins, known virulence factors, phage components, flagella and extracellular proteins in the vesicle fraction, and a low, negative correlation was found between transcript-, and protein enrichment. This result opposes the hypothesis that a significant degree of protein translation occurs in vesicles after budding. The abundance of viral-, and flagellar proteins in the vesicle fraction underlines the importance of purification during vesicle isolation.

## 1. Introduction

Extracellular vesicles (EVs) are membrane-bound bodies regularly secreted by Gram-negative bacteria (Listgarten and Lai, 1979). At large, EVs consist of the same proteins, RNAs, DNAs, metabolites and lipopolysaccharides as their originator cell, but some reports indicate that specific proteins are enriched (Haurat et al., 2011; McMahon et al., 2012). EVs display highly diverse characteristics in shape (McCaig et al., 2013), single-, or double membrane structure (Pérez-Cruz et al., 2013), and typically vary in diameter by an order of magnitude (20–200 nm) (Chatterjee and Chaudhuri, 2011). Several bacterial mechanisms have been proven to be associated with the secretion of EVs, such as biofilm formation, nutrient acquisition and secretion of virulence determinants into host cells (Kulp and Kuehn, 2010). For example, EVs inhibit the adhesion of the pathogen *Xylella fastidiosa* inside xylem vessels, enabling wider spread throughout host plants (Ionescu et al., 2014), and they arm *Vibrio cholerae* with a defense mechanism against bacteriophages (Reyes-Robles et al., 2018). Interestingly, EVs can also transfer membrane portions that contain phage receptor proteins, and in this way propagate susceptibility to certain phages (Ofir and Sorek, 2017). This raises the question whether some phages could induce production of EVs for this very purpose. Other findings indicate that certain plasmids may induce the production of EVs, and thereby facilitate their own spread (Erdmann et al., 2017).

EVs are known to contain mRNA and non-coding RNA (ncRNA), and it has been demonstrated that they can deliver their RNA cargo into eukaryotic cells (Dauros-Singorenko et al., 2018). While some inclusion of RNA is expected when volumes of the intracellular space is incorporated during vesicle formation, the mechanisms behind DNA inclusion in EVs are still unclear. When not undergoing chromosome replication, it is generally assumed that bacteria contain a similar quantity of any part of their chromosomes. The same is not necessarily true for vesicles, which could be secreted in order to communicate specific DNA sequences into the environment. While the genetic transfer capability of certain EVs is established, it is of interest to gain an overview of which DNAs and RNAs that are specifically enriched, and understand how these might affect their environment. The genetic content of EVs from several bacteria have previously been sequenced (Biller et al., 2014; Sjöström et al., 2015; Bitto et al., 2017), revealing that specific genome regions and transcripts are significantly more abundant than others. These data, however, were not quantitatively compared to coverage discrepancies in sequence data from the parent bacteria. It is not known whether the genetic content of vesicles is actively transcribed or translated after secretion, calling for a precise proteomic profile of the vesicles in question. Being that EVs have a lower surface area to volume ratio, intuition states that vesicles should contain a higher proportion of membrane-associated proteins than their parent cell, but they may also be enriched with non-membrane proteins of vesicle-specific function. The proteomes of EVs from several bacteria have been mapped previously, revealing that they include proteins from all subcellular locations, while mainly membrane proteins, such as those related to biofilm formation, virulence and antimicrobial resistance, are enriched (Altindis et al., 2014; Lagos et al., 2017).

Due to their compositional similarity to their parent and non-replicative nature, EVs have been proposed as vaccines against many pathogens (Acevedo et al., 2014), and is being used commercially against e.g., *Neisseria meningitidis* (Holst et al., 2009). As vaccine candidates, it is important to assess the capacity of EVs for genetic transfer, since they may potentially be administered in environments that frequently contain other pathogens, such as hospitals, aquaculture facilities or livestock farms. They are known to enable cross-species transfer of virulence genes, including antibiotic resistance (Yaron et al., 2000), which may call for some restrictions when it comes to strains, antibiotic markers and growth conditions that are fit for the production of EV-based vaccines. Furthermore, the production of vesicles carrying specific DNAs or RNAs may be of interest in therapeutic or microbiological applications, underlining the importance of identifying motifs or other attributes that may increase genetic enrichment. While some basic research on vesicles requires purification steps such as density gradient centrifugation, this may not be cost-efficient for some industrial or pharmaceutical purposes, e.g., vaccines meant for farmed fish. It is therefore of interest to analyse the complete EV isolate, so that any non-EV material included in a potential vaccine is not ignored.

The main aim of this work was to investigate the potential preferential inclusion of genetic material in EVs, using *V. cholerae* O395 mutant TCP2 (Mekalanos et al., 1983) as a model organism. *V. cholerae* is naturally competent when grown on chitin, an abundant material in its natural environment (Meibom et al., 2005), indicating that it maintains a level of interspecies genetic communication. Furthermore, *V. cholerae* continues to be a detrimental pathogen, meaning that the results may carry strain-specific medical relevance. The TCP2 mutant lacks a complete CTX*ϕ* bacteriophage, including the genes encoding both subunits of the cholera toxin that leads to the debilitating diarrhea associated with cholera. CTX*ϕ* is a temperate phage, and its genomic DNA may at times be present in wild-type O395 strains in quantities sufficient to overshadow other DNA fragments during sequencing. In addition, the two copies of toxin co-regulated pilin precursor gene *tcpA* are deleted, which could have given rise to excessive non-vesicular extracellular matter. The TCP2 genome harbors a streptomycin resistance gene, and the use of a selective medium minimizes the risk of interference by foreign DNA from potential contamination. The genome of *V. cholerae* is divided into chromosome 1 (ChI) at approximately 3.0 Mbp, and chromosome 2 (ChII) at 1.1 Mbp, which may give further insight in specific packaging of genetic material into the EVs (Sjöström et al., 2015). In general, ChI harbors the larger portion of essential genes, while ChII encodes a larger proportion of hypothetical proteins (Cameron et al., 2008). ChII includes a ~35 kbp K139 prophage (Reidl and Mekalanos, 1995), as well as a superintegron containing hundreds of seemingly species-specific gene cassettes (Rowe-Magnus et al., 1999), which may also be differentially included in EVs.

## 2. Methods and Materials

### 2.1. Cultures and Media

All cultures were grown using LB medium or LB agar at 37°C with 200 μg/mL streptomycin, and liquid cultures were grown at 100 rpm shaking. Frozen stock of *V. cholerae* was plated and grown overnight. Two 100 mL starter cultures were inoculated from plate colonies and grown overnight. Twenty-five milliliters of starter culture was added to each of four or eight 2.5 L volumes of medium, and grown overnight to late log phase, i.e., OD_600_ ≈ 1 (Figure S1), when contamination from lysed cells is minimal (Sjöström et al., 2015). The process was performed three times to yield independent biological triplicates (*n* = 3).

### 2.2. EV Isolation

The EV isolation protocol is largely the same as described for marine samples (Biller et al., 2014). A minimum of 10 L *V. cholerae* culture was centrifuged (using Andreas Hettich Bottles: 0551, part no. 4AJ-7900519) at 4,000 × *g* for 30 min, transferred to cleaned bottles and centrifuged at 4,000 × *g* for another 30 min. The bacterial pellets were kept at −20°C until DNA, RNA and protein isolation. The supernatant was filtered through 0.45 μm and then 0.2 μm filters (durapore cat no. HVLP14250 and qvwp14250), using a 142 mm filter holder (Millipore Corporation 01730) and a peristaltic pump. The volume was then concentrated to approximately 25 mL using a 100,000 NMWC hollow cartridge filter (GE Healthcare 56-4101-72) at 15 psi inlet pressure. Three hundred milliliters of PBS was added and this dilution was again concentrated to approx 150 mL. The volume was split in 10 mL aliquots and kept at −20°C (< 7 days) to reduce RNA degradation while performing replicates. The whole volume was filtered through a 0.45 μm filter and then ultracentrifuged at 100,000 × *g* for 2 h (SW 32 Ti rotor). The supernatant was discarded and another volume of filtrate was added before another centrifugation (100,000 × *g*, 2 h). The supernatant was discarded and the tubes were filled with PBS and centrifuged again (100,000 × *g*, 2 h). Each pellet was resuspended in approximately 1.5 mL PBS and kept at −20°C (< 7 days) to reduce RNA degradation until RNA isolation. This isolate is henceforth referred to as the extracellular vesicle fraction (EVf), and each of all three EVf replicates was used for DNA, RNA, and protein analysis, in order to minimize the effect of biological variations between the techniques. The protein content of the isolates was measured to be approximately 11.8 mg/mL. The samples were inspected by negative-stain electron microscopy to assess vesicle integrity and presence of contaminations. A ~ 100 μl portion of the bacterial pellet was resuspended in 5 ml PBS. This isolate is henceforth referred to as the whole cell fraction (WC).

### 2.3. DNA and RNA Preparation

Before DNA was isolated, the vesicle isolate was split in 100 μL aliquots, 4U DNase was added to each aliquot and incubated at 37°C for 30 min. Another 4U was added and the incubation repeated, before the DNase was inactivated at 75°C for 15 min. RNase was not deemed fitting before RNA isolation, due to the fact that RNases are not as easily inactivated as DNases, and excessive heat or chemical treatment could reduce the integrity of the vesicles or their RNA cargo before isolation. Furthermore, some secondary structures may provide certain RNAs with protection from RNAses, making the definite localization of these difficult (Blenkiron et al., 2016).

#### 2.3.1. DNA Isolation

All DNA quantification was performed using Qubit™ dsDNA HS assay kit (Thermo Fisher). For EVf-DNA isolation, 200 μL aliquots were treated with the Qiagen EZNA mini kit type 1, according to manufacturer's protocol, with the exception of adding four volumes of supernatant lysate to the same HiBind^®^ DNA Mini Column, and eluted in 30 μL kit-provided nuclease-free water. This kit preferentially isolates fragments under 45–50 kbp in length, with an effective cutoff at 150 kbp (Qiagen, 2012). Therefore, this kit was also used to isolate DNA from WC, to ensure similar conditions for both samples, and to avoid coverage lost to genomic DNA (gDNA). Similarly, 200 μL aliquots of WC were used for DNA isolation, as described for EVf-DNA above. The DNA isolation yielded 60–70 ng/μL for WC-DNA samples, and approximately 0.4–0.6 ng/μL for the EVf-DNA samples. This corresponds to approximately 1.75 ng DNA isolated per mg of protein in the EVf, and 3 ng EVf-DNA per liter bacterial culture. The samples were cleaned with Agencourt^®^ AMPure^®^ XP according to manufacturer's protocol. gDNA for genome assembly was isolated from the bacterial pellet with QIAGEN Genomic-tip 100/G according to manufacturers protocol.

#### 2.3.2. RNA Isolation

All RNA quantification was performed using Qubit™ RNA HS assay kit (Thermo Fisher). RNA was isolated from EVf with Allprep^®^ DNA/RNA Mini Kit (Qiagen cat. 80204) treating 200 μL aliquots as starting material, and adding 500 μL RLT buffer. Four to six of these volumes were passed through a single DNA column, and the flowthrough from 2 to 3 of these columns were treated according to protocol, and passed through a single RNA column. RNA was eluted in 30 μL RNase-free water, resulting in approximately 7 ng/μL. This corresponds to approximately 23 ng RNA per mg of protein in the EVf, or 40 ng EVf-RNA per liter bacterial culture. RNA from WC was isolated in the same manner, and yielded approximately 70 ng/μL. The EVf-RNA samples and 1 μg of the each of the WC-RNA samples were prepared with Truseq mRNA stranded kit (Illiumina), quality controlled with NGS kit (Agilent) on Fragment Analyser (ATII), and amplified with KAPA Library Quantification Kit (Roche), all according to manufactures protocol.

### 2.4. Sequencing and Analysis

DNA and RNA samples were sent to Norwegian Sequencing Centre to be sequenced on Illumina HiSeq 4000, 2x150 paired-end run with 350 bp insert size.

#### 2.4.1. Reference Genome Assembly

The 5.1 M gDNA reads were assembled *de novo* using SPAdes (v3.10.1). They were also aligned to an O395 reference genome (GenBank: GCA_000016245.1) with the burrows-wheeler aligner (BWA, v0.7.8)(Li and Durbin, 2010), resulting in 99.68% of the reads being successfully mapped. The alignment was inspected in Geneious (v10.1.3)^1^ to identify regions with no coverage. Four regions of zero coverage were identified, and replaced with the corresponding sequence data from the SPAdes assembly. After correction, the number of aligned reads increased with 2.6 k reads to 99.73%, with no gaps in coverage. The modified genome was annotated using RAST (Job# 492506) (Aziz et al., 2008). Putative phage genes were predicted using PHAST (Zhou et al., 2011).

#### 2.4.2. Coverage Analysis

The raw EVf-DNA and WC-DNA reads were trimmed using trim-galore (v0.3.3), aligned to the O395 TCP2 genome using BWA, and replicates removed using picard-tools (v2.10.4)^2^. Cuffdiff (through cufflinks v2.2.1) was used for enrichment analysis for DNA and RNA, analogously to an RNA differential expression analysis. Cuffdiff uses a genome annotation file for expression analysis, and since intergenic regions could be of interest, placeholder annotation files were created, assigning an identifier to every kbp of the genome. Ten such files were created with 100 bp offsets, with starting bp = 0, 100, 200…, in order to construct a sliding window enrichment table after enrichment analysis. The GC-content of each sliding window was calculated using bedtools (v2.17.0). The process was identical for RNA data, with the exception that duplicate reads were not removed, and the RAST-annotated genome was used for expression analysis. The enrichment of very abundant ncRNAs was also calculated using per-base coverage provided by bamtools (v2.4.0) (Barnett et al., 2011), normalizing ncRNA coverage by the average RNA read coverage over the full genome for each sample.

### 2.5. Protein Analysis

Protein was quantified using Pierce^TM^ BCA Protein Assay Kit (Thermo Scientific^TM^ 23225) according to manufacturer's protocol. In-solution digestion and liquid chromatography mass spectrometry (MS) was performed on EV and WC samples as previously described (Aqrawi et al., 2017), with the exception that database searches were performed on a protein database with 3920 entries constructed from the RAST-annotated *V. cholerae* TCP2 genome using transeq through EMBOSS (Rice et al., 2000) (v6.5.7). Data were analyzed using Scaffold (v4.8.4, Proteome Software Inc., Portland, OR). Peptide identifications were accepted if they could be established at greater than 95.0% probability by the Scaffold Local FDR algorithm, while protein identifications were accepted if they could be established at greater than 99% probability and contained at least 2 identified peptides. Using Scaffold, a *T*-test and multiple test correction with Benjamini–Hochberg was done using WC as fold change reference category. The subcellular location of each gene was found using PSORTb version 3.0.2 (Yu et al., 2010).

### 2.6. Electron Microscopy

Freshly formvar-coated 200 mesh grids were used for both negative stain- and immunogold electron microscopy, and all micrographs were captured with a JEOL 1400 plus microscope at 100 keV. For negative stain images, grids were placed on droplets of EVf for 1 min, washed 3 times on PBS for 1 min, and fixed on 4% paraformaldehyde (PFA) in PBS for 2 min. Finally, the grids were washed 10 times on H_2_O for 1 min, and stained on 4% uranyl acetate (UA) for 2 min. Excess UA was dried off using a filter paper, and the grids were left to dry for 10 min before imaging.

#### 2.6.1. Immuno-Gold Labeling

10 μL of EVf-sample was diluted in 12% gelatine at 37°C to a total volume of 200 μL. Thirty microliters droplets of the solution were placed on parafilm in room temperature to solidify for 10 min. The solid droplets were fixed in 4% PFA/PBS at 4°C overnight, then submerged in sucrose and cut into ~0.5 mm cubes, which were placed on silver rods and flash-freezed in liquid nitrogen. The samples were sectioned in a cryomicrotome at −120°C to a thickness of approximately 45 nm, and picked up using a loop dipped in 2% methyl cellulose (MC) at 4°C and transferred onto the grids. The grids were suspended on a droplet of PBS on ice for 3 min, and placed on a droplet of 1:1 Antibody (MAB030 anti-dsDNA clone BV16-13 from Sigma-Aldrich)/1% fish skin gelatine (FSG) for 50 min. The grids were then washed 5 times on PBS for 3 min, before being transferred to a droplet of 1%FSG/proteinA-gold for 20 min. The grids were washed 3 times on PBS for 3 min, followed by 10 times on H_2_O for 1 min. Finally, the grids were stained on 4% UA for 2 min, after which the grids were picked up with a loop, excess liquid removed and left to dry for 10 min before imaging.

## 3. Results and Discussion

### 3.1. Electron Micrographs Reveal DNA Associated With EVs From *V. cholerae*

**Figure 1:**
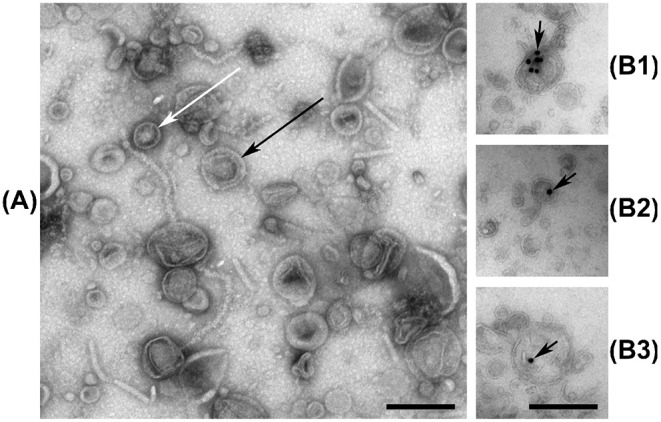
**(A)** Negative stain electron micrograph of the vesicular fraction of *V. cholerae*, with white and black arrows indicating examples of single-, and double membrane vesicles, respectively. Some filamentous structures are also visible. **(B1–B3)** Ultrathin-section electron micrographs of the vesicular fraction of *V. cholerae* labeled with anti-dsDNA antibodies and gold nanoparticles, indicated by black arrows. Black bars are 200 nm long.

Micrographs reveal that the extracellular fraction of *V. cholerae* contains both double- and single membrane vesicle structures (Figure 1A). Some of the visible filamentous structures are likely viral-, flagellar-, or pilin constructs, similar to previous observations (Kondo et al., 1993), but certain endogenous phages of *Vibrio* species can be difficult to distinguish from vesicles (Lorenz et al., 2016), making precise EM characterization of EV samples challenging. No complete tailed phage structures were visually confirmed in our samples. The ultrathin-section micrographs labeled with dsDNA antibodies reveal the presence of DNA within the outline of membrane vesicles (Figures 1B1–B3). The background labeling is insignificant, confirming that DNA in the EVf is largely confined within vesicular bodies or embedded in their membranes, although some DNA may still reside within phage particles too small to effectively access during thin-sectioning. This observation is similar to previous localization of DNA in vesicles from *Streptococcus mutans* (Zheng et al., 2009) and *Pseudomonas aeruginosa* (Bitto et al., 2017). The nature of double-membrane vesicle budding allows transport of cytoplasmic matter, including chromosomal DNA. However, regarding the secretion of single-membrane vesicles, intuition states that their interior should originate from the periplasmic space, and therefore not contain considerable quantities of DNA of chromosomal origin. Some unknown mechanism(s) may therefore transport DNA-fragments out into the periplasmic space before budding, or freely diffusing DNA may be absorbed into the periplasm from the extracellular space, before or after budding (Renelli et al., 2004; Mashburn-Warren et al., 2008; Seitz et al., 2014). A so far unreported explanation could be that the outer membrane may be shed during or after budding, due to perforations in the outer membrane, post-budding stress, or some other uncharacterized mechanism. A process reminiscent of this has been reported in the Gram-positive *Bacillus subtilis*, where vesicles form in prophage-induced holes in the peptidoglycan wall (Toyofuku et al., 2017).

### 3.2. EV-Associated DNA Is Characteristically Different From Whole Cell DNA

Sequencing of WC-DNA yielded 3.7–19.6M reads per sample, of which 97.0–99.3% were successfully mapped to the genome. Sequencing of EVf-DNA yielded 3.7–34.6M reads per sample, of which 97.2–98.6% were successfully mapped to the genome. The mapped read coverage reveals certain regions of the genome that are more abundant across all replicates, some characteristic to either EVf- or WC-samples (Figures S2A,B).

#### 3.2.1. Phage DNA

**Figure 2:**
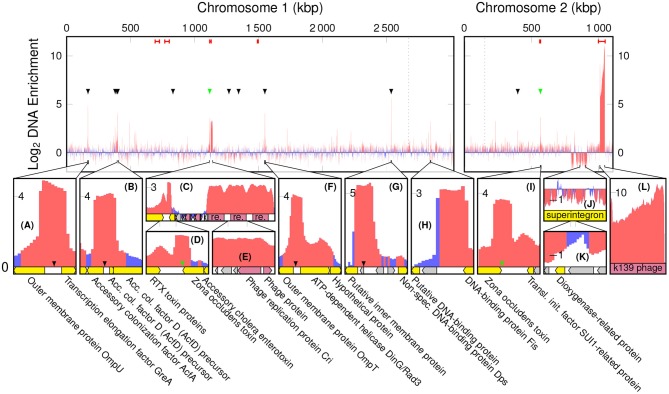
Log_2_ enrichment of kbp partitions of the *V. cholerae* genome according to cuffdiff, red denoting statistically significant enrichment. Red bars at the top are regions in which PHAST recognized phage genes, black arrowheads indicate a ToxR binding motif of the form AAAAAAMATMAAA, and green arrowheads indicate a toxbox region (TTTTGAT heptad repeat). Dotted lines are replication origo of the chromosomes. The bottom panels **(A**–**L)** display regions of interest, in which yellow tracks denote characterized genes, gray hypothetical, and red phage components.

The highest peak in coverage in EVf-DNA spans the K139 prophage, and in WC-DNA spans the superintegron (Figure S2B). The ratio between sequencing coverage of ChII and ChI in WC did not differ significantly when including or excluding the K139 prophage (Figure S2C), while the difference was considerable for EVf (Figure S2D). This stands testament to the abundance of the K139 prophage DNA in EVf, and suggests that the prophage DNA escapes the bacteria into extracellular DNase-protected states shortly after synthesis. Cuffdiff determined several significantly enriched kbp partitions of the genome (Figure 2). The K139 prophage was the most enriched DNA in EVf (Figure 2L) (some kbp partitions up to 2,300×, and on average 246×), and insert size estimation in Geneious revealed a high number of reads mapped approximately -34.5 kbp apart in ChII, suggesting that the phage is undergoing a significant amount of rolling-circle amplification. Within the ~700–800 kbp region of ChI, PHAST detected two inverted copies of a Mu-like prophage. Between these prophages, a ~43,200 bp region encodes a series of genes associated with metabolism and regulation, such as *hns* (Ramisetty et al., 2017), *hisD* (Chiariotti et al., 1986), *cspD* (Yamanaka et al., 2001), and *atoS* (Theodorou et al., 2012). Mu is a dsDNA bacteriophage (Bukhari and Ambrosio, 1978), but its lack of DNA enrichment suggests that it is not a replicating phage under the present growth conditions. Some other partial prophages were detected by PHAST; a triple repeat of phage replication protein Cri (Figures 2C,E), as well as remnants of the partially deleted CTX*ϕ* phage (Figures 2D,I) were highly enriched in EVf, while not nearly as dramatic as the K139 phage genome. Although K139 DNA is enriched in EVf, the phage structures may not be abundant in electron micrographs, as the DNA density of the capsid is ~425 Mbp/μm3 (assuming a roughly spherical capsid 54 nm across and 35 kbp genome; Reidl and Mekalanos, 1995), while the average DNA density the of *V. cholerae* interior is ~22 Mbp/μm3 (assuming a cylindrical cell shape with hemispherical ends, 1.6 μm long and 0.4 μm in diameter, harboring a 4.1 Mbp genome; Baker et al., 1983). Additionally, since many vesicles may not contain DNA, only a few complete capsids could potentially amount to the same DNA quantity as hundreds of vesicles.

#### 3.2.2. ToxR Binding Motifs

There are a number of non-prophage regions that are highly enriched in EVf. The most enriched of these sequences harbor known binding sites for ToxR (Figures 2A–D,F,G,I), a transcriptional regulator located in the cytoplasmic membrane. Interestingly, an association between EVs and proteins regulated by ToxR has been reported previously (Altindis et al., 2014). ToxR is known to regulate e.g., transcription of *ompU, ompT*, and *acfA* by binding to an upstream binding motif (AAAAAANATNAAA) (Kazi et al., 2016), and the upstream regions of these three genes were all highly enriched (Figure 2A,B,F). Additionally, the most enriched DNA region of ChI (Figure 2G) contains this motif, upstream of a putative membrane protein. This protein, a homolog of lysoplasmalogenase YhhN (Jurkowitz et al., 2015), could potentially also be under the regulation of ToxR. All the sites in the genome matching this motif were identified (Figure S3A), and we observe the consensus motif for the most enriched (>4×) regions, AAAAAAMATMAAA (M signifying an amine; A or C) (Cornish-Bowden, 1985). DNA mapped to this refined binding motif was significantly enriched (~5.6×) in EVf (Figure S3B), while the most enriched (~10.6×) unambiguous motif was AAAAAAAATAAAA. ToxR also regulates *ctxAB* by binding to a “toxbox” region (TTTTGAT tandemly repeated 3–8 times) (Miller et al., 1987), and while both copies of *ctxAB* is deleted from the TCP2 genome, their toxbox regions are intact and significantly enriched, directly downstream of the gene encoding Zona occludens toxin (*zot*) (Figures 2D,I). Aside from *zot*, RTX cytotoxin proteins were enriched (Figure 2D), which is similar to a previous report demonstrating that DNA encoding cytotoxin ExoS was abundant in EVs from *P. aeruginosa* (Bitto et al., 2017). The enrichment in EVf of sequences containing ToxR binding motifs may have its explanation in that ToxR is membrane-associated, and its binding can thus provide a direct connection to the cytoplasmic membrane, which in turn may be enriched in the vesicles. Additionally, ToxR binding could protect the motif itself from DNases, which may also contribute to enrichment (Goss et al., 2013).

#### 3.2.3. Restriction Sites

One of the few highly enriched regions that do not contain the ToxR binding motif, harbors a 12× repeat of the motif 5′-TCTAGAATCC-3′ (Figure 2H). This sequence provides a number of restriction sites for restriction enzymes XbaI and TfiI, between a putative DNA-binding protein and DNA-binding protein Fis. Restriction sites could potentially increase the probability of DNA inclusion in vesicles, as loose ends and smaller DNA fragments may be more likely to enter protrusions in the bacterial membrane during vesiculation.

#### 3.2.4. Superintegron

Although a less prominent phenomenon, some regions of DNA were significantly depleted in EVf. Most notably, relatively lengthy parts of the superintegron structure in ChII (Figures 2J,K). This region is high in coverage in WC-samples, which stands in contrast to previous *V. cholerae* sequence data from fecal samples (Sealfon et al., 2012). This could be explained by the fact that in the present study a different DNA isolation kit was used, tailored for shorter fragments. The discrepancy may be due to the mobile nature of transposons (Marin and Vicente, 2013), as excised fragments may be enriched in the WC sample with our kit.

#### 3.2.5. GC-Content

**Figure 3:**
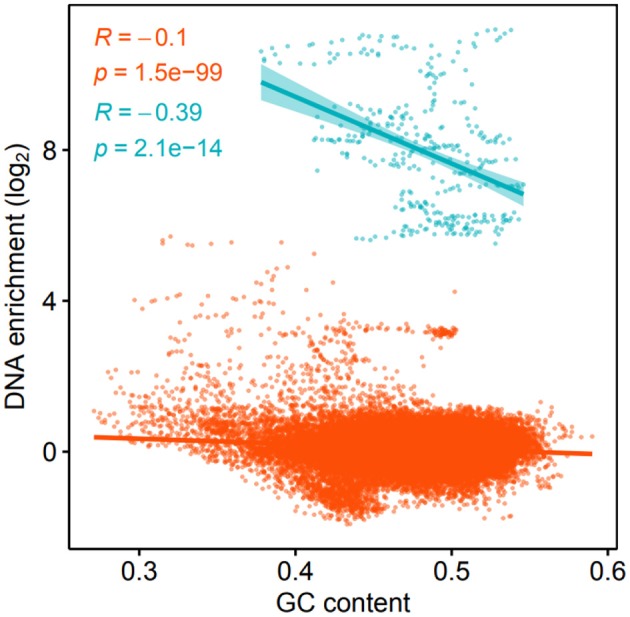
Log_2_ DNA enrichment of kbp partitions of the *V. cholerae* genome and GC-content. Blue color denotes windows within the K139 prophage, while orange denotes windows of the rest of the genome, respectively.

The enrichment of kbp genome partitions and their GC-content was found to correlate negatively, both for the K139 phage DNA (*p* = 2.1·10^−14^) and the rest of the genome (*p* = 1.5·10^−99^) (Figure 3). There are several factors that may be contributing to this phenomenon; firstly, AT-rich regions of the genome are more accessible (Gomes and Wang, 2016), and a higher density of protein binding to these regions could increase their association with the bacterial membranes, or provide protection against DNases. Protein binding sites are preferentially located between protein coding regions (Ishihama, 2010), and the intergenic GC content of the TCP2 genome is only 41.4% while it is 48.5% for protein-coding regions, according to the RAST-annotation.

### 3.3. EV-Associated RNA Is Characteristically Different From the Whole Cell Transcriptome

**Figure 4:**
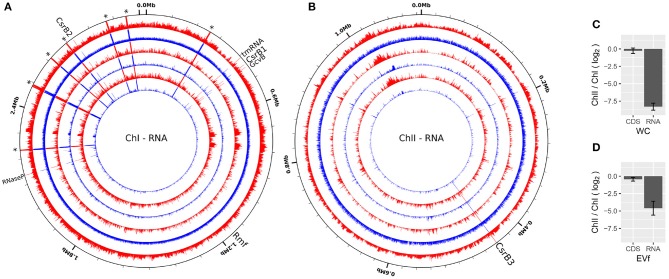
RNA sequencing coverage over ChI **(A)** and ChII **(B)** of *V. cholerae* from EVf (red) and WC (blue) from three biological replicates (Log scale). A selection of ncRNAs are named, while asterisks denote ribosomal RNA. Made using circleator (v1.0.0) (Crabtree et al., 2014). **(C,D)** Average RNA coverage ratio ChII/ChI in WC and EVf, for total RNA, or protein coding sequences (CDS) only.

**Figure 5:**
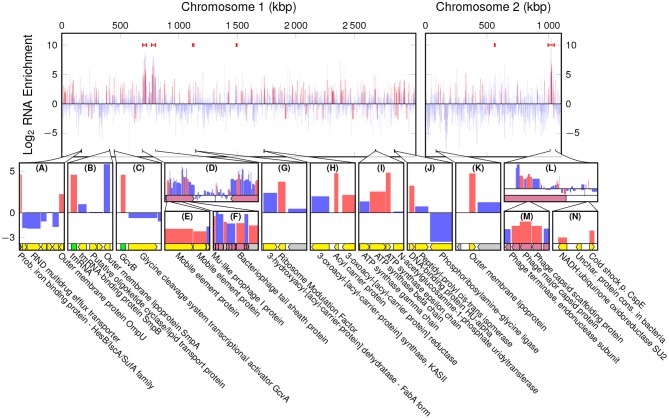
Log_2_ Enrichment of annotated RNAs in EVf, according to cuffdiff, red denoting statistically significant enrichment. Red bars are the regions in which PHAST recognized phage genes. The bottom row **(A–N)** displays regions of interest, in which yellow tracks signify characterized genes, gray hypothetical, red phage components, and green ncRNA.

The EVf-RNA sequencing resulted in 23.6M–78.2M reads per sample, whereof 99.5–99.9% were mapped to the genome. WC-RNA sequencing resulted in 23.7–96.3M reads per sample, of which 96.8–99.6% were mapped to the genome. The mapped read coverage reveals that similar to the DNA data, certain regions display higher coverage across all replicates, some being characteristic to either EVf- of WC-samples (Figures 4A,B). According to cuffdiff, a total of 214 annotated transcripts were significantly enriched in the EVf, distributed irregularly across both chromosomes (Figure 5).

#### 3.3.1. Ribosomal RNA

A large portion of sequencing reads stem from ribosomal RNA, and since they are all encoded in ChI, transcriptional products from ChII are very scarce in comparison. Counting only read coverage mapped to protein coding sequences (CDS), the difference is not that pronounced, but EVf maintains a significantly lower coverage in ChII than in ChI (Figures 4C,D). Results from previous RNA sequencing of *V. cholerae* reveal that genes on ChI are more frequently transcribed than those on ChII during growth in rich media, although the difference is less pronounced when grown in rabbit intestine (Xu et al., 2003). This upregulation of certain genes on ChII in intestine led to the suggestion that ChII-genes may be more important during infection, e.g., in response to nutritional stresses. The difference in ChII/ChI coverage ratio when counting total RNA or only CDS is smallest in EVf, implying that rRNA is depleted in the extracellular fraction.

#### 3.3.2. Functional-, and Non-coding RNA

A series of conserved functional and non-coding RNAs are visible as peaks in mapped read coverage, most notably RNase P type A, tmRNA and CsrB1 in ChI, and CsrB3 in ChII (Figures 4A,B). CsrB3 is the most abundant form of RNA from in ChII, and in this study amounts to ~70% of RNA mapped to this chromosome for both EVf and WC. The most consistently enriched of these are tmRNA and RNase P (Figure S4). The protein component of RNAse P has been found to be anchored to the inner membrane in *Escherichia coli*, which could partly explain its abundance in EVf (Miczak et al., 1991). The abundance of tmRNA has been observed in the extracellular milieu of *E. coli* previously (Ghosal et al., 2015), and this enrichment could partly be due to its binding to elongation factor Tu, commonly found in vesicle preparations (Blenkiron et al., 2016). sRNAs CsrB1, CsrB2, CsrB3 (Nguyen et al., 2018) and GcvB are also significantly enriched (Figure S4). CsrB sRNAs have been found to take part in virulence and biofilm formation, and to be regulated by quorum sensing (Lenz et al., 2005). In *E. coli* CsrB RNAs is known to antagonistically regulate the action of carbon storage regulator CsrA (Liu et al., 1997). CsrA binds to the 5' untranslated regions (UTRs) of mRNA, recognizing GGA motifs in apical loops of RNA secondary structures. These motifs are also present on CsrB sRNAs, effectively sequestering the CsrA protein, allowing translation of the formerly inhibited mRNAs (Dubey et al., 2005; Duss et al., 2014). Interestingly, CsrA was only detected in WC, suggesting that other factors may be responsible for the enrichment of CsrB sRNAs in EVf. GcvB is known to regulate several genes in *E. coli*, e.g., RNA polymerase sigma S (RpoS), which enables the bacteria to survive under lower pH (Jin et al., 2009). This sRNA is dependent on binding to Hfq for its regulatory effect (Pulvermacher et al., 2009), and this may contribute to its enrichment, as Hfq preferentially associates with the bacterial membrane (Diestra et al., 2009).

#### 3.3.3. Phage mRNAs

While enrichment in EVf-DNA was characterized by enrichment of CTX*ϕ* and K139 phage genes (Figures 2C,L), the inverted Mu-like prophages in ChI are more pronounced in RNA enrichment (Figure 5D). Mu-like phage DNA was not particularly enriched, but several of its transcripts are, including tail sheath and capsid genes. Similarly, mRNA for many structural proteins of phage K139 were significantly enriched (Figures 5L,M). Enrichment of phage RNA associated with host-relationship modulation has previously been observed in EVs from *Salmonella enterica* (Malabirade et al., 2018).

#### 3.3.4. Bacterial mRNAs

Cuffdiff found that a probable iron binding protein mRNA and *ompU* were enriched (Figure 5A), the latter of which was also associated with a coverage peak in DNA. Other highly enriched genes from ChI were *rmf* (Terui et al., 2010), *acpP* (Kutchma et al., 1999), *atpD, atpC* (Dunn et al., 2000), and *hupA* (Mart́ınez et al., 2015) (Figures 5G–K). Interestingly, the other subunit of DNA-binding protein HU, *hupB* (Mart́ınez et al., 2015) was enriched as well (Table S1). The HU dimer is in *E. coli* known to bind with high affinity to the mRNA encoding RpoS, and the ncRNA DsrA (Balandina et al., 2001), which in turn regulates transcription by overcoming the silencing effect of DNA-binding protein H-NS on the expression of RpoS. HU has also been found to regulate virulence of *Vibrio parahaemolyticus* (Phan et al., 2015). ChII harbors a number of enriched genes, e.g., an Outer membrane protein mRNA (Figure 5K), and *cspE* (Figure 5N). Cold-shock protein E (CspE) is regularly expressed at 37°C, and was originally identified as a multicopy suppressor of a temperature-sensitive chromosome partition mutant (Yamanaka et al., 1994).

**Figure 6:**
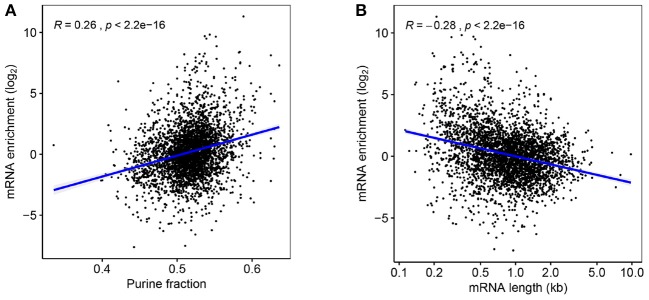
**(A)** Fold-change enrichment of mRNAs in EVf, plotted against purine content. **(B)** mRNA enrichment according to transcript length.

The etiology of mRNA enrichment in vesicles is not yet fully understood, but there are presumably three important mechanisms that are most important for this phenomenon; half-life, location, and size. Firstly, mRNA synthesis is likely low in EVs compared to living bacteria, meaning that mRNA with longer half-lives will be enriched in vesicles over time. It has been reported that mRNAs in thermophile prokaryotes are biased toward purine tracts, indicating that it provides thermostability (Paz et al., 2004), and in this study, a positive correlation was indeed found between purine content and enrichment of mRNAs in EVf (Figure 6A). Although the samples have been subjected to the lowest possible thermal stress during isolation, it could still be that some level of purine-dependent degradation has occurred in the duration of filtration and isolation. It has been observed that the uracil content of membrane mRNAs is higher in prokaryotes than in eukaryotes (Prilusky and Bibi, 2009), a pattern that coincides with the differing mRNA turnover requirements of the two branches of life. To check whether the purine–enrichment correlation in our cultures could be caused by more complex underlying sequence dependencies, the pearson correlation coefficient was calculated between occurrences of all possible RNA nonuplets and mRNA enrichment. Some of these yielded very low p-values (Figure S5), especially for specific purine tracts, such as NNNAAGNNN, NNNNGAAGA, and NNAAGAAGA. This indicates some level of sequence dependency of the mRNA enrichment, which in turn could be due to increased stability, or perhaps affinity to localizing-, or protecting, biomolecules. Curiously, the sextuplet that correlates most significantly with mRNA enrichment is AGAAGA, the *in-vivo* binding motif for the human mRNA splicing protein TRA2B (Änkö and Neugebauer, 2012), which shares significant RNA-binding domain similarity with an uncharacterized RNA-binding protein in *V. cholerae* (NCBI Reference Sequence: WP_001884322.1, 2018). In addition, this protein is likely a cytoplasmic membrane protein, according to PSORTb.

The second mechanism that could give rise to mRNA enrichment in EVf is indeed the location, affected by potential affinity to membranes or membrane proteins, such as the signal recognition particle (SRP), which is associated with the membrane through the SRP receptor (Akopian et al., 2013). Through SRP, mRNAs could be co-localized with their protein products during translation, which in turn could place them close to the cytoplasmic membrane. While this may be true for certain mRNAs, no significant enrichment was found for genes destined for any location in this study (Figure S6). However, mRNAs have been found to localize to the membrane in *E. coli* independent of translation (Nevo-Dinur et al., 2011), meaning some motifs or secondary structures may increase an mRNAs affinity toward the membrane regardless of their protein product. Even though a number of RNA motifs correlate positively with enrichment in this study (Figure S5), the folding nature of RNA makes their protein interactions more complex than what may be deduced from the primary structure alone.

A third mechanism likely to affect enrichment is size, as a portion of the vesicles are smaller than many mRNA sequences. While vesicles can be as small as 20 nm, typical RNAs can vary in size from ~7 to 33 nm for 0.7–8.9kbp sequences, respectively (Borodavka et al., 2016). In accordance with this restriction, the correlation between mRNA length and enrichment in EVf was negative and statistically significant (Figure 6B). This result is similar to findings on the size-dependent inclusion of plasmids in EVs (Tran and Boedicker, 2019), and suggests that vesicles maintain a significant size exclusion bias for genetic cargo. This correlation holds for annotated gene length, and is independent of the lengths of the actual isolated RNA fragments, information that is lost in the sequencing protocol.

### 3.4. The EV Proteome Is Enriched in Periplasmic-, Membrane-, and Extracellular Proteins

The results from genetic sequencing prompted us to map the proteomic profile of EVs in order to asses a possible correlation between enrichment of genes and protein products. By LC-MS, a total of 1312 proteins were detected, 670 of which in common while 222 and 420 were exclusively detected in the EVf or WC, respectively.

#### 3.4.1. Subcellular Location

Proteins associated with the extracellular milieu, outer membrane and periplasm are significantly enriched in EVf compared to cytoplasmic proteins (Figure 8). The variation within each category is considerable, which indicates that the composition of proteins in each category is different from WC to EVf. For instance, one could expect all the outer membrane proteins to be enriched along with the membrane itself, but their variation from a high degree of enrichment to a high degree of depletion suggests that the mechanisms of protein enrichment are more complex than random budding of the membranes. The specific enrichment or depletion of certain membrane proteins has been observed before (Lee et al., 2008; Aguilera et al., 2014), supporting the hypothesis that vesicles are not simply random membrane blebs, but at least somewhat specifically constructed.

#### 3.4.2. mRNA–Protein Correlation

**Figure 7:**
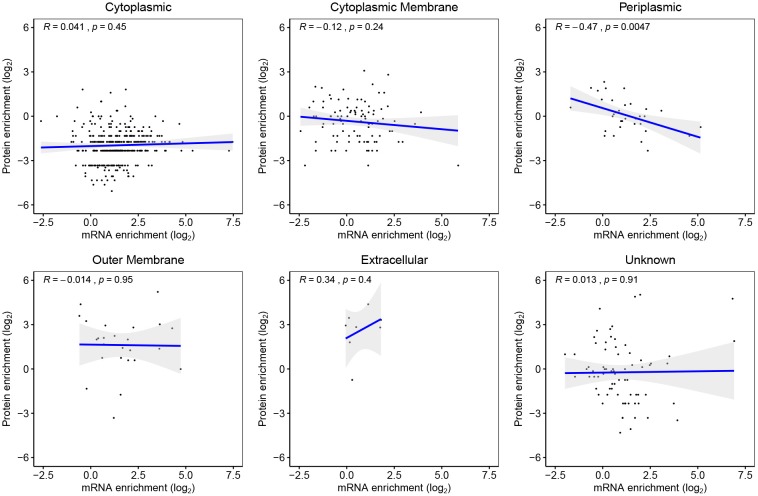
Genes detected as both mRNA and their protein products in both EVf and WC, sorted by their predicted subcellular location. Periplasmic proteins yielded the only statistically significant regression analysis, showing a negative correlation between mRNA and protein enrichment.

580 genes were detected both as mRNA and their protein products in both WC and EVf. Only genes destined for the periplasm demonstrated a statistically significant mRNA–protein enrichment correlation, that being negative (Figure 7). These results suggest that post-budding translation of vesicle-associated mRNA is low. This does not, however, refute the possibility that mRNA cargo could be translated in a potential target cell for the EVs. Worth to mention in this context is that no genes were found as both protein and mRNA in one fraction uniquely, with the sole exception of Soluble cytochrome b562, a protein that was found in only one of three EVf replicates.

#### 3.4.3. RNA-Binding Protein

**Figure 8:**
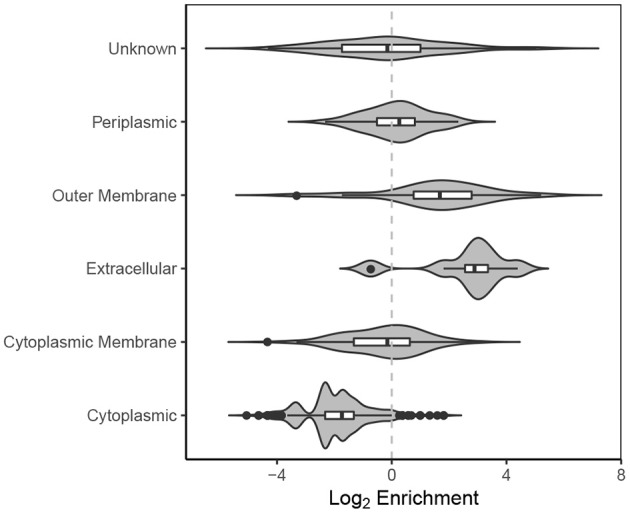
Violin plot of log_2_ enrichment of proteins in EVf relative to WC and their cellular location.

An interesting EVf-enriched protein in the context of RNA enrichment is the methionine ABC transporter substrate-binding protein (Table S2B). This membrane protein, also called ProQ, has been shown in *S. enterica* and *E. coli* to bind to sRNA and mRNA, increasing their stability (Smirnov et al., 2016). This protein is enriched >8× in EVf, which is well-beyond the standard deviation of the general enrichment of cytoplasmic membrane proteins (Figure 8). The presence of this protein could in part responsible for the enrichment of certain sRNAs or mRNAs; for instance, it is known to bind to and stabilize *cspE* mRNA (Holmqvist et al., 2018), which was enriched >16× in EVf. This protein could act as a link between certain RNAs and the membrane, and depending on its RNA-binding affinities, be decisive for the composition of the RNA cargo of vesicles. Several CsrB sRNAs are abundant in EVf, and while these are known to bind to CsrA protein, no CsrA protein was found in the EVf. This is surprising, as one could expect that biomolecules with demonstrated affinity to each other would be co-localized. A possible explanation could be that these RNAs also bind to ProQ (Holmqvist et al., 2018), contributing to their enrichment.

#### 3.4.4. Flagella and Phage Proteins

A number of flagellar and extracellular proteins were the most abundant in the extracellular fraction compared to whole cells. Of the 48 known flagella-associated proteins in the O395 proteome, a total of 39 were detected in the samples. 9 were found only in the bacterial fraction, including regulators such as FleN and FleQ, motor switch proteins such as FliG and FliM, and synthesis proteins such as FliS and FlgN. 17 flagellar proteins were uniquely detected in the EVf, mostly structural components such as FliD, FlaG, FlaF, FlgL, FlgK, FlgG, FlgC, FlgB, FlgF, but also the biosynthesis protein FlhF, L- and M-ring proteins FlgH and FliF, and the hook-length control protein FliK (Kim and McCarter, 2000). 13 proteins encoded by the K139 prophage were detected by LC-MS; 10 unique to EVf, 1 unique to WC, and 2 detected in both samples; tail fiber and major capsid protein, enriched 5.7× and 27×, respectively. This not nearly as dramatic as the enrichment of the prophage DNA, which according to cuffdiff was 249× on average. This indicates enrichment of phage DNA in other DNase-protected states than within phage particles, such inside- or embedded in the membrane of EVs, or possibly in other phage capsid structures. Structural components of both the Mu-like phage in ChI and K139 in ChII were enriched in the EVf, while no complete phage structures were visible in the micrographs, as has been observed before (Reidl and Mekalanos, 1995). This could imply that the K139 tail structures may be utilized by *V. cholerae* as a tailocin (Ghequire and De Mot, 2015), but this hypothesis needs testing. The high presence of flagellar and phage components in EVf sheds light on the importance of purification when working with EVs. This may be especially important in their application of vaccine candidates, when certain epitopes are of interest, and protein quantification may be used as a dose measurement. Since phages are co-isolated due to their similar sizes, they can pose a significant problem when working on vesicles specifically. One solution to this is density gradient centrifugation, but as this process may take 16 h (Olaya-Abril et al., 2014), mRNA stability is a concern.

#### 3.4.5. Virulence Factors

Beyond flagellar-, and phage constituents, there were many proteins highly enriched and depleted in EVf (Table S2). Expectedly, among the most enriched were proteins associated with membranes or the periplasmic space. Furthermore, many of the abundant proteins uniquely detected in EVf (Table S2A) are associated with virulence, e.g., OmpK (Ningqiu et al., 2008), TraF-related protein (also called type IX secretion protein SprF/PorP) (Laanto et al., 2014), colonization factor AcfA (Hughes et al., 1995), and OmpT (Provenzano and Klose, 2000). Similarly, among the most enriched proteins detected in both samples are virulence factors such as TonB (Abdollahi et al., 2018), OmpU (Provenzano et al., 2001), OmpV (Liu et al., 2017), haemagglutinin biogenesis protein MshL (Hsiao et al., 2006), hemolysin Vcp, and outer membrane protein LapE of the TolC family (Smith et al., 2018). A surprising observation is the enrichments of OmpK, OmpU and OmpV, which mirror the high enrichment of their mRNAs, and may point to a degree of co-translational localization. Many of these virulence factors (e.g., AcfA, OmpU, MshL, TolC) have been observed in the proteome of EVs from *V. cholerae* previously (Altindis et al., 2014) and stands testament to the potential of EVs to modulate host–pathogen relationships. For instance, vesicles with hemolytic proteins could be used as a remote agent to damage host cells, increasing the nutritional value of the bacterial environment.

#### 3.4.6. Iron Transport Proteins

There are several proteins associated with iron uptake that are enriched significantly in EVf. For instance, a ferrichrome-iron receptor was only found in EVf, while heme-, and sidophore receptors HutA (Henderson and Payne, 1994) and IrgA (Wyckoff et al., 2015) were highly enriched (Table S2). These proteins mediate iron uptake, which has some metabolic implications. If vesicles could contribute to iron depletion of the surroundings, this would supposedly affect the bacteria negatively unless they have mechanisms for vesicle re-absorption.

#### 3.4.7. Antimicrobial Resistance

While the bacteria need to effectively absorb iron compounds in low-iron conditions, antimicrobials are preferably effluxed out. Several relevant acriflavin-resistance proteins are highly enriched in EVf (Tables S2A,B). For instance, VexH, VexB, and VexD were enriched while VexK was only detected in EVf. These are members of the AcrB/AcrD/AcrF protein family, known multidrug efflux transporters (Buckley et al., 2006). The AcrB protein is located in the inner membrane, where it interacts with the outer membrane protein TolC through periplasmic protein AcrA. Deletion of *acrB* or *tolC* is associated with hypersensitivity to a range of antibiotics in *S. enterica* (Buckley et al., 2006). Another enriched protein related to resistance is YcfM, also known as LpoB, which has been identified as an activator of penicillin-binding-protein PbpG (Jean et al., 2015). A second protein from the *ycf* operon, YcfL, was among the most abundant only detected in EVf. Its location on the same operon could imply an association with drug resistance, but little is known about this lipoprotein (Alam et al., 2013). The presence of efflux pumps on vesicles could mean that the bacteria gain some benefit from them being largely void of antimocrobials, which does not contradict the hypothesis that they can be re-absorbed.

#### 3.4.8. Adhesion and Biofilm Formation

Two other proteins previously detected in EVs from*V. cholerae* are RmbA and RmbC (Altindis et al., 2014). These were both enriched in EVf (Tables S2A,B) and have been found to be important for biofilm formation. RbmA is a extracellular protein that forms tandem fibronectin type III (FnIII) folds (Giglio et al., 2013), and is required for rugose colony formation and biofilm structure integrity (Fong et al., 2006). RbmC has been recognized as a hemolysin and a central agent in biofilm and pellicle formation, perhaps binding to carbohydrates in the extracellular matrix (Fong and Yildiz, 2007). It is secreted and localized on the cell surface (Teschler et al., 2015). The association of EVs with biofilm formation has been established for some time (Kulp and Kuehn, 2010), and have been found to be a major structural part of biofilm in *Staphylococcus aureus* (He et al., 2019).

#### 3.4.9. Protein Depletion in EVf

A thorough analysis of proteins depleted in EVf is beyond the main scope of this study, but some interesting observations deserve comment. As expected, cytoplasmic proteins were significantly depleted in EVf (Figure 8), but many DNA-binding proteins stemming from genes that were enriched in EVf (Table S1) were depleted considerably (Tables S2B,C). For instance, HU-α and HU-β are depleted by 70 and 80%, while *hupA* and *hupB* mRNA were some of the most enriched mRNAs in EVf. Similarly, H-NS protein is depleted by 80% in EVf, while *hns* mRNA was enriched >7× on average. Ssb is depleted by 80% while *ssb* mRNA was enriched >11×, and transcription termination factor Rho is only detected in WC while *rho* was enriched >3× in EVf. While the mechanisms behind this is unknown, it could be that these mRNAs are more stable than the average, or that they somehow localize to the membrane, maybe by protein binding. DNA-binding protein HU has been found to bind to both tmRNA and RNase P RNA in *E. coli* (Macvanin et al., 2012), which were both enriched in EVf.

## 4. Conclusion

The present study is the first exploratory work that directly compares the DNA, RNA and protein content of *V. cholerae*, to that of its extracellular vesicles. Many interesting observations shed light on the general composition of the vesicle fraction, including their cargo of DNA, functional RNA, mRNA, RNA-binding proteins and virulence factors. While the observed nucleic acid enrichment patterns seem to have possible explanations in membrane protein binding, specific investigations are required to confirm the specific mechanisms involved. For instance, a future research objective is to investigate the effect of ToxR and its binding sites on the enrichment of DNA in vesicles. An alternative study could be on the effect of ProQ or other RNA-binding proteins on RNA enrichment. A series of known virulence proteins were enriched in EVf, which is to be expected, as the host-pathogen interaction is modulated in part by the outer membrane proteins of *V. cholerae*. Tail tube and tail fiber proteins of several prophages were abundant in EVf, while no complete phage structures were visible in electron microscopy. This could imply function as tailocins, but further research is required to assess this hypothesis. A discrepancy between transcript enrichment and the enrichment of their protein products in EVf argues against significant translation occurring after vesicle isolation, but the effects of mRNA turnover in this context is unclear. A future prospect is therefore to investigate the effects of translation-, transcription-, and RNase inhibitors on the bacterial culture before vesicle isolation. An interesting observation in the EVf proteome is that enrichment of genes for several DNA-binding proteins contrasted the depletion of their protein product, while for a number of outer membrane proteins, mRNA enrichment mirrored the proteomic profile. This could suggest either a certain level of co-translational mRNA localization, or perhaps differing turnover requirements for the transcripts of DNA-binding- and membrane proteins. A research prospect is to locate these mRNAs within the living bacteria, and identify the affinities of RNA-binding membrane proteins. If the mechanisms behind the observed enrichment in DNA, RNA and protein are well-characterized, this would be a big step on our way to produce customized vesicles. In turn, this could greatly expand our possibilities when it comes to the utilization of vesicles as vaccines or vessels for gene-, or drug delivery.

## Data Availability Statement

The datasets generated for this study can be found in NCBI, accession numbers: PRJNA586749 and PRJNA587223.

## Author Contributions

PL has planned and performed most of the experiments and contributed in writing of the manuscript. AK has planned and performed some of the experiments and contributed in writing of the manuscript. HW-L had contributed in planning of the experiments and in writing of the manuscript.

### Conflict of Interest

The authors declare that the research was conducted in the absence of any commercial or financial relationships that could be construed as a potential conflict of interest.

## Footnotes

^1^*Geneious: Suite of Molecular Biology and NGS Analysis Tools*. Available online at: https://www.geneious.com/

^2^*Picard Tools: Command Line Tools for Manipulating High-Throughput Sequencing (HTS) Data*. Available online at: http://broadinstitute.github.io/picard
