# Supplementary material for: Vesicles From Vibrio cholerae Contain AT-Rich DNA and Shorter mRNAs That Do Not Correlate With Their Protein Products
Source: Front Microbiol. 2019 Nov 22;10:2708. doi: 10.3389/fmicb.2019.02708 (PMC6883915; doi:10.3389/fmicb.2019.02708)
Supplement: Supplementary file 1 [file Presentation_1.pdf]

SUPPLEMENTARY FIGURES

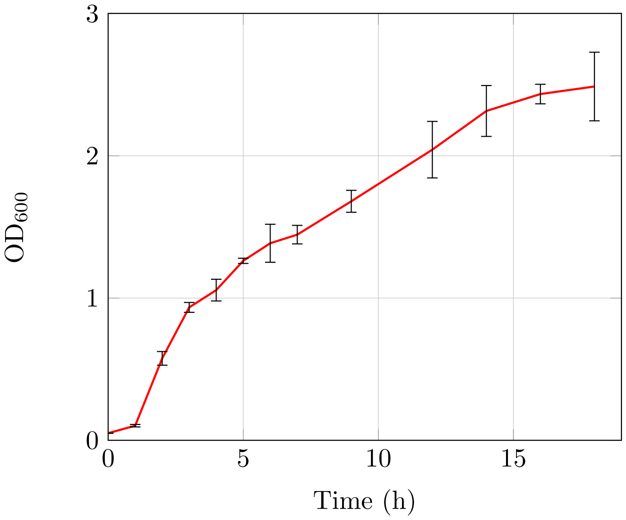

Figure S1. Typical growth curve of *V. cholerae* TCP2 in 200 mL LB volume at 37 °C. EVs were harvested at $OD_{600} \sim 1$.

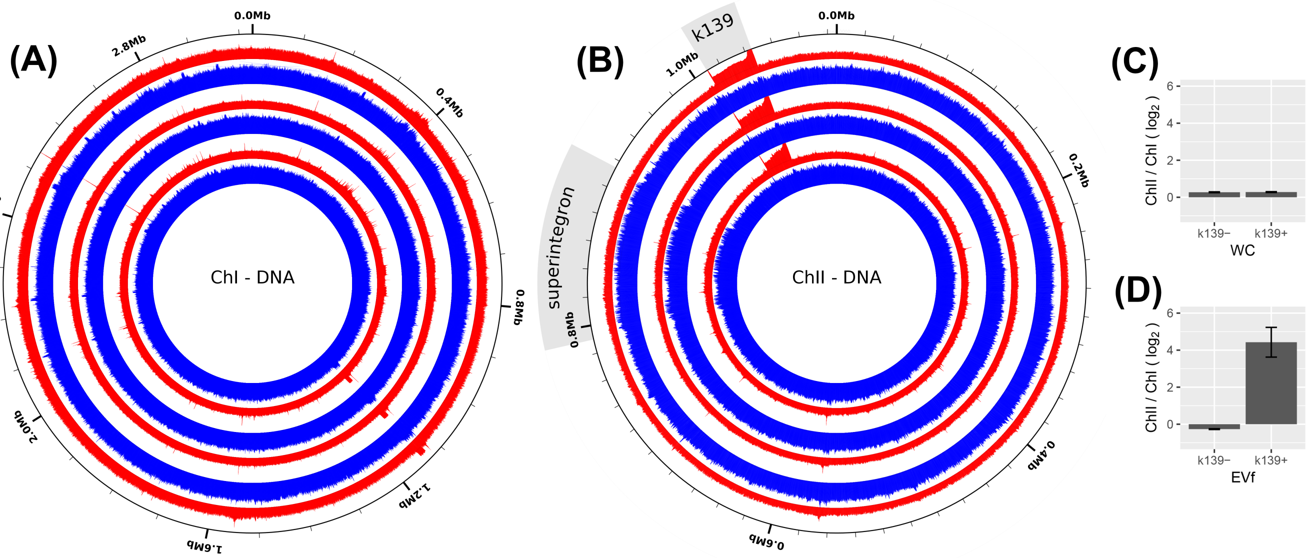

Figure S2. Normalized DNA sequencing coverage over ChI (**A**) and ChII (**B**) of *V. cholerae* from EVf (red) and WC (blue) from three biological replicates (Log scale). The K139 prophage and superintegron region are emphasized in ChII. Made using circleator (v1.0.0).[113] **(C)** and **(D):** average coverage ratio ChII/ChI in WC and EVf, respectively, K139+ and K139- denotes the inclusion and exclusion of the K139 prophage from quantification, respectively.

Ch	Position	Log2-enrichment	Significant	Sequence
1	2536386	5.51	yes	AAAAAAAATCAAA
1	166948	4.65	yes	AAAAAAAATAAAA
1	1549066	4.07	yes	AAAAAACATAAAA
1	399488	4.01	yes	AAAAAAAATAAAA
1	379445	2.64	yes	AAAAAAAATAAAA
2	397564	2.29	yes	AAAAAAAATAAAA
1	392965	2.02	yes	AAAAAACATAAAA
1	366166	1.09	yes	AAAAAACATTAAA
1	1268254	0.88	yes	AAAAAACATCAAA
1	1343873	0.69	no	AAAAAACATAAAA
1	831062	0.38	no	AAAAAAAATCAAA
1	1833342	0.19	no	AAAAAACATGAAA
1	1759734	0.06	no	AAAAAATATTAAA
	consensus	>2		AAAAAAMATMAAA

(A)

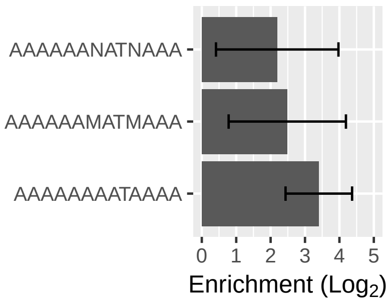

(B)

Figure S3. **(A)** Positive motif hits for ToxR binding site AAAAAANATNAAA and enrichment (Log_2) in EVf of kbp genome partitions centered upon them. Log_2 enrichments greater than 2 are highlighted in gray. **(B)** Average enrichment for kbp partitions centered on AAAAAANATNAAA motifs and selected derivatives.

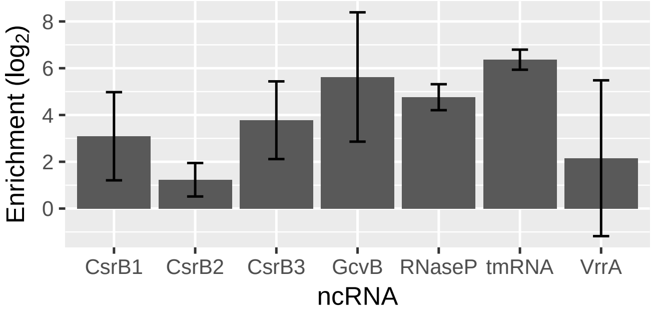

Figure S4. Average enrichment of ncRNAs in EVf (normalized by total mapped RNA reads), according to per-base coverage data.

	Motif	Estimate	p-value	mRNAs
3n	NNNAAGNNN	0.295	3.82e-71	3860
4n	GAAGNNNNN	0.295	7.37e-71	3545
5n	NNNNGAAGA	0.237	8.29e-46	2637
6n	NNNAGAAGA	0.186	1.15e-28	1220
7n	NNAAGAAGA	0.137	3.97e-16	623
8n	NAAGCGCCA	0.148	1.57e-18	133
9n	CUAUCAGCU	0.124	1.98e-13	16
A^-	NNGUUCNNN	0.162	7.51e-22	3128
G^-	ANNNCNAAN	0.188	2.93e-29	3566
R^-	CNCCCNUUC	0.080	2.44e-06	113

Figure S5. Pearson correlation estimates between mRNAs enrichment and occurrences of selected nonuplets per base of transcript length. 3n–9n denotes a minimum unambiguous length of 3 to 9 bases, respectively. A^-, G^- and R^- denotes adenine, guanine, and purine-free nonuplets, respectively. The last column is the number of mRNAs that harbors at least one copy of the nonuplet.

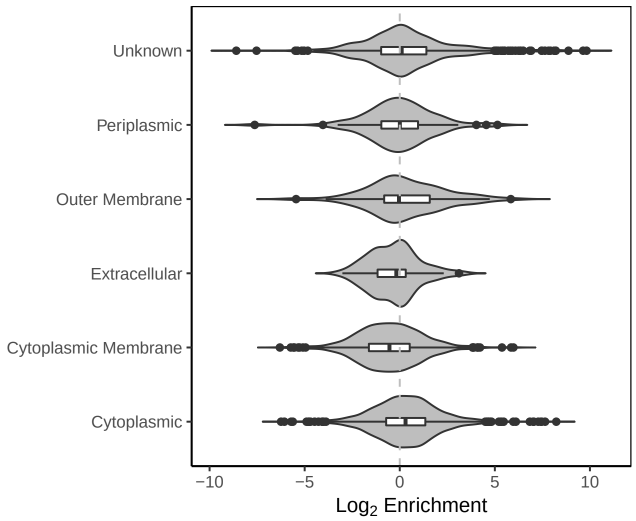

Figure S6. Violin plot of the enrichment of mRNAs in EVf compared to WC, and the subcellular location of their protein products.

Table S1. Selected non-phage transcripts of high enrichment in *V. cholerae* or its extracellular milieu, sorted from highest to lowest enrichment. **(A)** Transcripts only found in EVf. **(B)** Transcripts found in both EVf and WC, highest enriched in EVf on the top, and highest enriched in WC on the bottom. **(C)** Transcripts only detected in WC, sorted from lowest to highest abundance.

	Chr.	C_{WC}	C_{EVf}	Enrich. ($\log_2$)	Name
(A)	ChI	0	10.8	inf	FIG01200900: hypothetical protein
	ChII	0	7.7	inf	FIG01200711: hypothetical protein
	ChI	0	7.1	inf	FIG01199699: hypothetical protein
	ChI	0	4.6	inf	Glutaredoxin-related protein
	ChI	0	4.3	inf	DNA-binding protein inhibitor Id-2-related protein
	⋮				
(B)	ChII	0.48	16.7	5.14	Periplasmic maltose/maltodextrin ABC transporter MalE
	ChI	1.40	39.5	4.82	ATP synthase epsilon chain
	ChI	2.80	76.5	4.77	Acyl carrier protein
	ChII	6.06	161.1	4.73	Outer membrane lipoprotein
	ChI	0.77	18.6	4.59	Prob. Fe binding protein - HesB/IscA/SufA family
	ChI	0.66	12.8	4.28	Outer membrane protein OmpV
	ChI	2.38	42.0	4.14	Aspartate ammonia-lyase
	ChI	0.89	15.0	4.08	Fumarate reductase subunit D
	ChII	2.04	33.4	4.03	Cold shock protein CspE
	ChI	0.98	15.9	4.02	FIG01200169: hypothetical protein
	ChII	1.28	19.1	3.90	Acetate kinase
	ChI	0.44	6.4	3.85	Outer membrane protein OmpK
	ChI	1.88	22.8	3.60	Integration host factor beta subunit
	ChI	0.92	10.4	3.50	DNA-binding protein Fis
	ChI	1.34	14.8	3.46	Single-stranded DNA-binding protein
	ChI	4.54	43.17	3.25	DNA-binding protein HU-alpha
	ChI	2.40	13.09	2.45	DNA-binding protein HU-beta
	ChI	10.87	51.5	2.24	Outer membrane protein OmpU
	⋮				
	ChI	59.2k	10.1k	-2.56	Large Subunit Ribosomal RNA
	ChI	59.1k	10.0k	-2.56	Large Subunit Ribosomal RNA
	ChI	59.1k	10.0k	-2.56	Large Subunit Ribosomal RNA
	ChI	59.4k	10.0k	-2.57	Large Subunit Ribosomal RNA
	ChI	59.2k	10.0k	-2.57	Large Subunit Ribosomal RNA
	ChI	59.4k	10.0k	-2.57	Large Subunit Ribosomal RNA
	ChI	59.3k	10.0k	-2.57	Large Subunit Ribosomal RNA
	ChI	58.0k	9.52k	-2.61	Large Subunit Ribosomal RNA
	⋮				
(C)	ChI	378.2	0	-inf	tRNA-Lys-TTT
	ChI	440.9	0	-inf	tRNA-Ile-GAT
	ChI	443.3	0	-inf	tRNA-Leu-TAG
	ChI	449.4	0	-inf	tRNA-Met-CAT
	ChI	455.4	0	-inf	tRNA-Asn-GTT
	ChI	513.8	0	-inf	tRNA-Ile-GAT
	ChI	513.9	0	-inf	tRNA-Asn-GTT
	ChII	516.8	0	-inf	tRNA-Gly-TCC
	ChI	519.1	0	-inf	tRNA-Ile-GAT
	ChI	714.2	0	-inf	tRNA-Met-CAT
	ChI	1128.7	0	-inf	tRNA-His-GTG
	ChI	1362.1	0	-inf	tRNA-His-GTG

Table S2. Proteins of high abundance in *V. cholerae* or its extracellular milieu. **(A)** Proteins only found in EVf, sorted from highest to lowest abundance. **(B)** Proteins found in both EVf and WC, highest enriched in EVf on the top, and highest enriched in WC on the bottom. **(C)** Proteins only detected in WC, sorted from lowest to highest abundance.

	Chr.	C_{WC}	C_{EVf}	Enrich. ($\log_2$)	Name
	ChI	0	28.7	inf	FIG01200881: hypothetical protein
	ChI	0	19.7	inf	Outer membrane protein OmpK
	ChI	0	19.3	inf	YcfL protein: an outer membrane lipoprotein
	ChII	0	15.3	inf	TraF-related protein
	ChII	0	14.3	inf	FIG01200406: hypothetical protein
	ChI	0	12.3	inf	Hemolysin-related protein RbmC
(A)	ChII	0	11.3	inf	FIG01199739: hypothetical protein
	ChI	0	9.7	inf	Accessory colonization factor AcfA
	ChII	0	8.7	inf	FIG01199666: hypothetical protein
	ChI	0	8.7	inf	Outer membrane protein OmpT
	ChI	0	8.3	inf	Ferrichrome-iron receptor
	ChI	0	8.0	inf	LPS-assembly lipoprotein RlpB precursor (Rare lipoprotein B)
	ChI	0	1	inf	Acriflavin resistance protein (VexK)
	⋮				
	ChI	1.33	49.7	5.21	Long-chain fatty acid transport protein
	ChII	0.67	14.3	4.39	TonB-dependent heme and hemoglobin receptor HutA
	ChI	1.00	17.3	4.09	Lipoprotein YcfM - part of a salvage pathway of unknown substrate
	ChI	1.3	16.0	3.58	TonB-dependent receptor / Enterobactin receptor IrgA
	ChI	3.0	30.0	3.32	Hemolysin-related protein Vcp
	ChI	0.7	6.3	3.25	MSHA biogenesis protein MshL
	ChI	1.3	11.3	3.09	Methionine ABC transporter substrate-binding protein
	ChI	1.3	10.3	2.94	Type I secretion system - outer membrane component LapE
	ChI	0.7	4.7	2.81	Lipoprotein nlpI precursor
	ChI	7.3	51.3	2.81	Outer membrane protein OmpU
	ChI	6.7	45.0	2.77	Outer membrane protein OmpV
	ChI	1.3	8.7	2.70	RbmA protein
	ChI	1	4.7	2.23	RND multidrug efflux transporter - Acriflavin resistance protein (VexH)
(B)	Ch2	0.7	2.7	2.00	Acriflavin resistance protein
	ChI	2.7	5.3	1.00	RND multidrug efflux transporter - Acriflavin resistance protein (VexB)
	Ch1	8	12	0.58	Acriflavin resistance protein (VexD)
	⋮				
	ChI	52.7	16.7	-1.74	DNA-binding protein HU-alpha
	ChI	8.0	1.67	-2.32	Single-stranded DNA-binding protein
	ChI	14.7	3.3	-2.32	DNA-binding protein HU-beta
	ChII	18.7	1.0	-4.32	Phosphomannomutase
	ChII	12.7	0.7	-4.32	4-alpha-glucanotransferase (amylomaltase)
	ChI	14.7	0.7	-4.32	Predicted dye-decolorizing peroxidase (DyP) - YfeX-like subgroup
	ChI	13.7	0.7	-4.32	Tyrosyl-tRNA synthetase
	ChI	13.3	0.7	-4.32	Cysteinyl-tRNA synthetase
	ChI	14.3	0.7	-4.32	Membrane alanine aminopeptidase N
	ChI	16.3	0.7	-4.64	Phosphopentomutase
	ChI	17.3	0.7	-4.64	Catalase
	ChI	20.3	0.7	-5.06	3,4-dihydroxy-2-butanone 4-phosphate synthase
	⋮				
	Ch1	8.7	0	-inf	Carbon storage regulator CsrA
	Ch2	10.7	0	-inf	N-acetylglucosamine regulated methyl-accepting chemotaxis protein
	Ch2	11.0	0	-inf	Methyl-accepting chemotaxis protein
	Ch1	11.3	0	-inf	1-deoxy-D-xylulose 5-phosphate synthase
	Ch1	12.0	0	-inf	Malonyl CoA-acyl carrier protein transacylase
(C)	Ch1	12.7	0	-inf	Glycogen synthase ADP-glucose transglucosylase
	Ch1	13.0	0	-inf	Phosphoglucosamine mutase
	Ch1	13.3	0	-inf	Transcription termination factor Rho
	Ch1	14.7	0	-inf	Methionine aminopeptidase
	Ch1	16.3	0	-inf	Uroporphyrinogen III decarboxylase
	Ch2	16.3	0	-inf	Oxygen-insensitive NAD(P)H nitroreductase
	Ch1	19.0	0	-inf	Alanine dehydrogenase
